# Comparing watershed afforestation and natural revegetation impacts on soil moisture in the semiarid Loess Plateau of China

**DOI:** 10.1038/s41598-018-21362-5

**Published:** 2018-02-14

**Authors:** Zongping Ren, Zhanbin Li, Xiaolu Liu, Peng Li, Shengdong Cheng, Guoce Xu

**Affiliations:** State Key Laboratory of Eco-hydraulics in Northwest Arid Region, Xi’an University of Technology, Xi’ an, 710048 China

## Abstract

Two contiguous watersheds in the Loess Plateau in China that differed in the way their vegetation had been restored—afforestation or natural revegetation—differed in their consumption of soil moisture: the afforested watershed consumed more soil moisture, although the difference was significant only in wet years. Yet, both the afforestation and natural revegetation did not induce the soil desiccation in the study area. In the afforested watershed, soil moisture was depleted even beyond a depth of 100 cm, whereas in the grassland (natural revegetation), the depletion was confined to a layer less than 60 cm deep. Rainfall in the growing season accounted for 46–60% of the variation in soil moisture in the 0–60 cm layer in the grassland, but only 22–39% of that in the forest land. Overall, afforestation is the better option for the Loess Plateau only in areas where the annual rainfall is more than 500 mm. In any attempt at revegetation, the choice of tree species and planting densities should match the carrying capacity of the region’s water resources.

## Introduction

Soil moisture is an essential component of the hydrological cycle^1^, particularly in arid and semi-arid regions, where it is fundamental to ecosystem sustainability^2,3^. According to the water balance equation, soil moisture is co-determined by rainfall, evapotranspiration, and run-off^4^. Vegetation, however, can impact soil moisture by intercepting rainfall with leaves^5^, by buffering infiltration and run-off through litter^6^, and by regulating water uptake through roots^7^. Therefore, the relationship between vegetation and soil moisture is critical to research in eco-hydrology^8–11^.

The Loess Plateau in China covers 6.4 × 10^5^ km², and is among the most ecologically fragile areas in the world^12^. The average annual rainfall in the region ranges from 150 mm in the north-west to 800 mm in the south-east, and annual evaporation, from 1400 to 2000 mm^13^. Because of the sparse vegetation, loose soil, and intense and heavy rains, the annual sediment discharge into the Yellow River can be as high as about 1.6 billion tonnes, which makes the region one of the most eroded in the world^14^. To mitigate soil erosion and to improve ecosystem services in the region, the Chinese government implemented a series of measures to increase the vegetation cover of the region in the past few decades, including the ‘Grain for Green’ programme, which was launched in 1999^12^. Between 1999 and 2013, vegetation cover on the Loess Plateau increased by 59.6% and the sediment discharge into the Yellow River in 2013 was only about 0.2 billion tonnes^14^.

However, this large-scale restoration of vegetation cover has also aggravated water scarcity, gradually leading to soil desiccation in many places on the Loess Plateau^2,13,15,16^. Concerned with the increasing shortage of water, many researchers sought to examine the relationship between revegetation and soil moisture on the Loess Plateau^17–21^ and found that exotic tree species and high-density planting intensify local depletion of soil moisture and therefore considered natural revegetation to be the better option for maintaining the water resources of the region^2,6,11,13^. However, these studies were based mostly on short-term data (typically 3 years or less^17,20,22,23^) on soil moisture: long-term (more than 10 years) data for the region on soil moisture remain scarce^11^, a shortcoming that makes it difficult to investigate the relationship between vegetation type and soil moisture across years with differing amounts of rainfall^24^. Understanding the effect of the pattern of rainfall on that relationship helps in optimizing the management of vegetation in the region.

It was against this background that the present study was set up, with the following objectives: (1) to elucidate the long-term effects of afforestation and natural revegetation on soil moisture; (2) to measure the inter- and intra-annual variation in soil moisture under afforestation and natural revegetation; (3) to investigate the differences between afforestation and natural revegetation in terms of soil moisture as influenced by different rainfall patterns; and (4) to explore the relationship between rainfall and soil moisture as affected by the type of vegetation.

## Methods

### Study site

**Figure 1:**
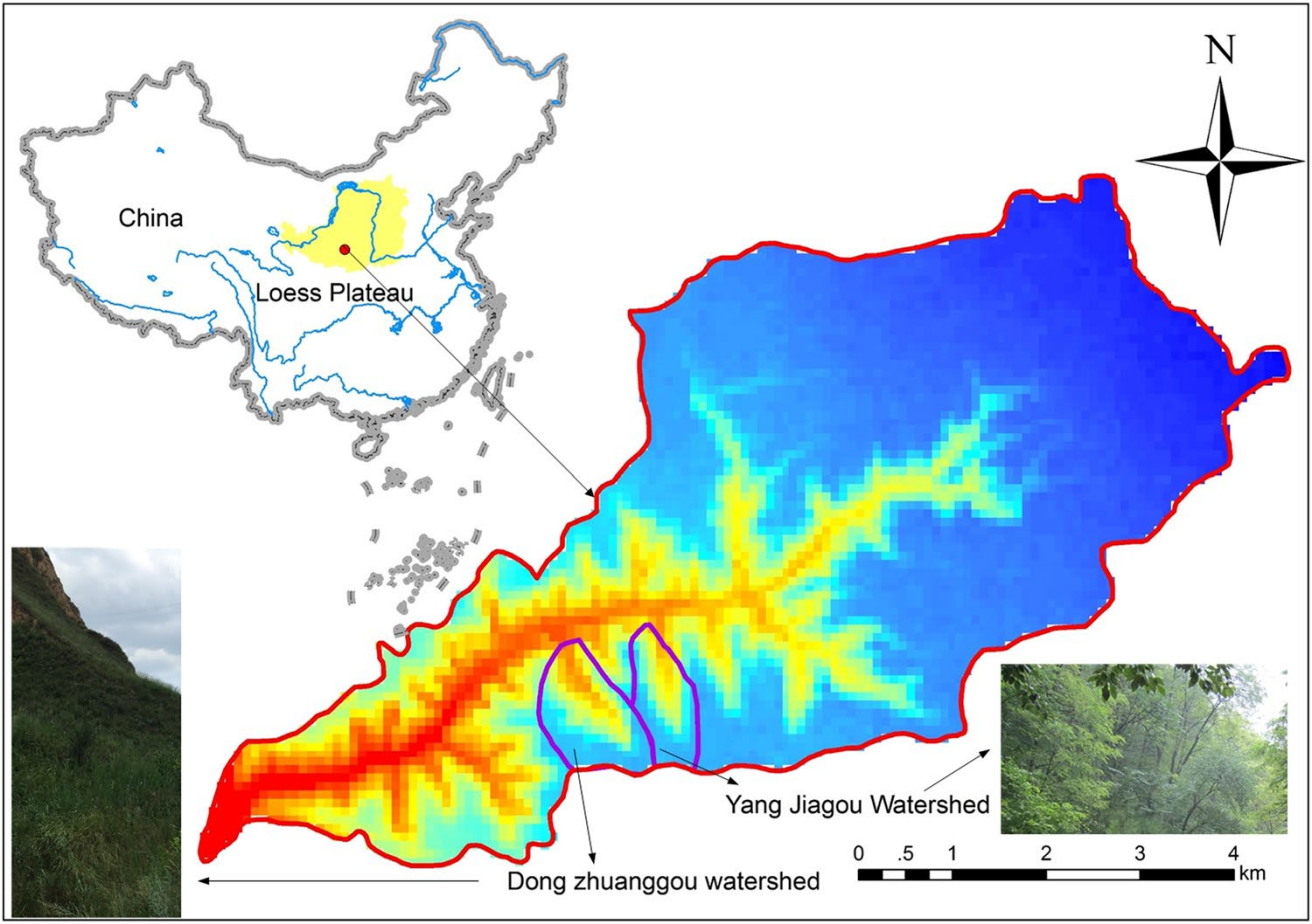
Location of the study site. The map was generated using ArcMap Version 10.0 (http://www.esri.com/); the DEM map of Nanxiaohe watershed was download from Geospatial Data Cloud website for free (http://www.gscloud.cn/).

The research was conducted in the Nanxiaohe basin (107°37′E, 35°42′N; 36.3 km²), approximately 7 km to the west of Qingyang city, in Gansu province, China. The study site is part of the Xifeng Soil and Water Conservation Station, established in 1951 by the Chinese government. The station itself comes under the Yellow River Conservancy Commission of the Ministry of Water Resources. The Nanxiaohe basin is the basin of a tributary of the Jing River, which flows through the central and southern parts of the Loess Plateau (Fig. 1). The site has a mean annual rainfall (1981–2014) of 515 mm, more than 80% of which is received from May to October; a mean annual evaporation of about 1500 mm; and a mean annual temperature of 9.2 °C (1980–2010 data for both parameters). The study area includes landforms that are typical of the gully region of the Loess Plateau, and elevations range from 1050 m to 1423 m. The soils are largely loessal and form a layer about 250 m thick on average. The soil texture is silt-loam and soil pH is approximately 8.4^25^. The natural biomes at the site are deciduous broad-leaved forests, for which the climax vegetation consists of stands of *Quercus liaotungensis*^26^.

The Nanxiaohe basin has two small and contiguous watersheds, namely Yangjiagou (YJG) and Dongzhuanggou (DZG), which were selected to compare the effects of soil and water conservation on different measures of restoring vegetation. The Yangjiagou watershed, 1.5 km long and covering 0.87 km², was afforested mainly with *Robinia pseudoacacia* during 1954–1958. The Dongzhuanggou watershed, 1.6 km long and covering 1.15 km², was allowed to recover its natural vegetation as part of the restoration efforts since 1954 and is now covered primarily by grasses such as *Arundinella hirta*, *Agropyron cristatum*, and *Artemisia argyi*. Owing to these two different approaches to restoration followed over more than 60 years, the two small watersheds offer two completely different vegetation landscapes^27^.

### Data sources and analysis

Data on soil moisture content (SMC) of the YJG and DZG watersheds from 1981 to 1994 were collected from the Xifeng Soil and Water Conservation Station. However, for reasons that could not be ascertained, the data had two gaps, namely from 1987 to 1988 in YJG and from 1987 to 1989 in DZG. Soil samples were taken from six layers, namely 0–10 cm, 10–20 cm, 20–40 cm, 40–60 cm, 60–80 cm, and 80–100 cm, using a drill, in the early, middle, and late parts of each month of the region’s growing season (May to October). The moisture content was determined by the oven-drying method, measured gravimetrically, and expressed as a percentage of the dry weight of the soil. Rainfall data for YJG from 1981 to 2014 were also collected from the Xifeng Soil and Water Conservation Station. Since the two watersheds are small and contiguous, the same rainfall data were used for both the watersheds.

### Rainfall classification

Based on the rainfall and its deviation from the mean, the years from 1981 to 1994 were divided into three categories, namely ‘normal’ years (rainfall within 10% of the average for 1981–2014), ‘dry’ years (90% of the average or less than that), and ‘wet’ years (110% of the average or more than that) (Table 1).

**Table 1:** Categories of years based on annual rainfall: 1981–1994.

Category	Rainfall (mm)	Mean (mm) ± standard deviation	Years
Normal	463–566	501 ± 30	1984, 1985, 1989, 1993, 1994
Dry	<463	401 ± 53	1982, 1986, 1991
Wet	>566	633 ± 34	1981, 1983, 1990, 1992

### Statistical analysis

The mean SMC for the growing season was calculated to represent the SMC for the sampling year. Independent *t*-tests were used to evaluate separately the differences in the mean annual SMC at different soil layers between YJG (referred to as the forest land from now on) and DZG (referred to as the grassland from now on). One-way analysis of variance (ANOVA) was used for evaluating the differences in the mean monthly SMC. The homogeneity of the variances among the groups was assessed by Levene’s test. Data screening found no significant difference for homogeneity of variance. The normal distribution test of the values of SMC was conducted by Shapiro-Wilk Test. Pearson’s correlation was used for examining the associations between annual rainfall, rainfall during the growing season, and SMC in each layer within each watershed. A general linear model analysed the impact of rainfall during the growing season on SMC of different layers in the forest land and grassland. The differences were evaluated at 5% significance level. All statistical analysis was conducted using SPSS ver. 16.0 (SPSS Inc., Chicago, USA).

### Data availability

The data that support the findings of this study are available from Xifeng Soil and Water Conservation Station of Yellow River Conservancy Commission of China but restrictions apply to the availability of these data, which were used under license for the current study, and so are not publicly available. Data are however available from the authors upon reasonable request and with permission of Xifeng Soil and Water Conservation Station of Yellow River Conservancy Commission of China.

## Results

### General characteristics of soil moisture content in profile

In the forestland, the mean annual SMC of the entire soil profile (0–100 cm) was 14.7% with a range of 10.0–17.8%; the value was maximum in the top layer (0–10 cm) and minimum in the deepest layer (80–100 cm). The coefficient of variation (CV) of SMC for each layer was between 14.5% and 23.6%, indicating moderate variation. The mean annual SMC in each layer ranged from 12.6% to 18.9% and the median, from 12.1% to 18.0%. Moreover, both the mean and the median values decreased with depth (Table S1).

Compared to the forestland, the mean annual SMC of the entire soil profile (0–100 cm) in the grassland was higher (17.7%; range, 14.5–23.2%). SMC was the highest in the 40–60 cm layer and the lowest in the 10–20 cm layer, and the CV for each layer was between 11.8% and 17.1%, also indicating a moderate variation. The mean annual SMC in each layer varied from 17.1% to 18.2% (median, 16.2–18.0%). In addition, both the mean and the median values in the upper layers (depth, 10–60 cm) were generally lower than those in the deeper layers (depth, 60–100 cm) (Table S1).

**Figure 2:**
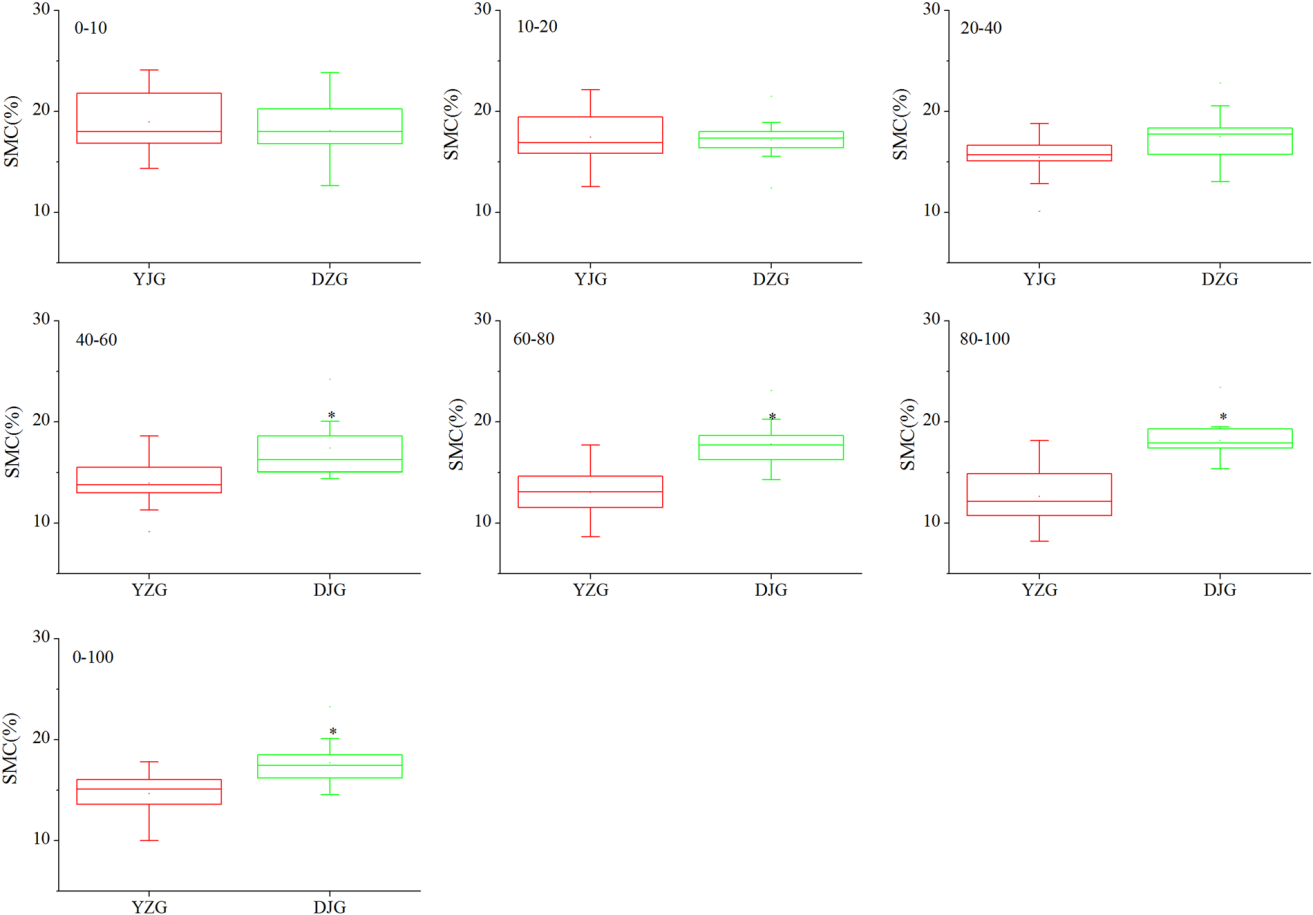
Mean annual soil moisture content at different depths in Yangjiagou (forest land) and Dongzhuanggou (grassland) watersheds. Mean values for 12 years for the forest land and for 11 years for the grassland.

In the forest land, SMC of the 0–10 cm and the 10–20 cm layers was slightly higher than the corresponding values in the grassland (P > 0.05). At depths greater than 20 cm, however, SMC in the forest land were lower than those in the grassland (Fig. 2); the *t*-test showed that SMC below 40 cm in the forest land was significantly lower than that in the grassland (P < 0.05) (Table 2). Overall, SMC across the profile in the forest land was significantly lower than that in the grassland (P < 0.05) (Fig. 2).

**Table 2:** Independent sample *t*-tests for mean annual soil moisture content at different depths in Yangjiagou (forest land) and Dongzhuanggou (grassland) watersheds.

Depth (cm)		0–10	10–20	20–40	40–60	60–80	80–100	0–100
*t*-test forequality of means	t	0.64	0.27	−1.99	−3.12	−4.71	−5.04	−3.26
	Sig.	0.53	0.79	0.06	0.005	0.000	0.000	0.004
	df	21	21	21	21	21	21	21

### Annual changes in soil moisture content

**Figure 3:**
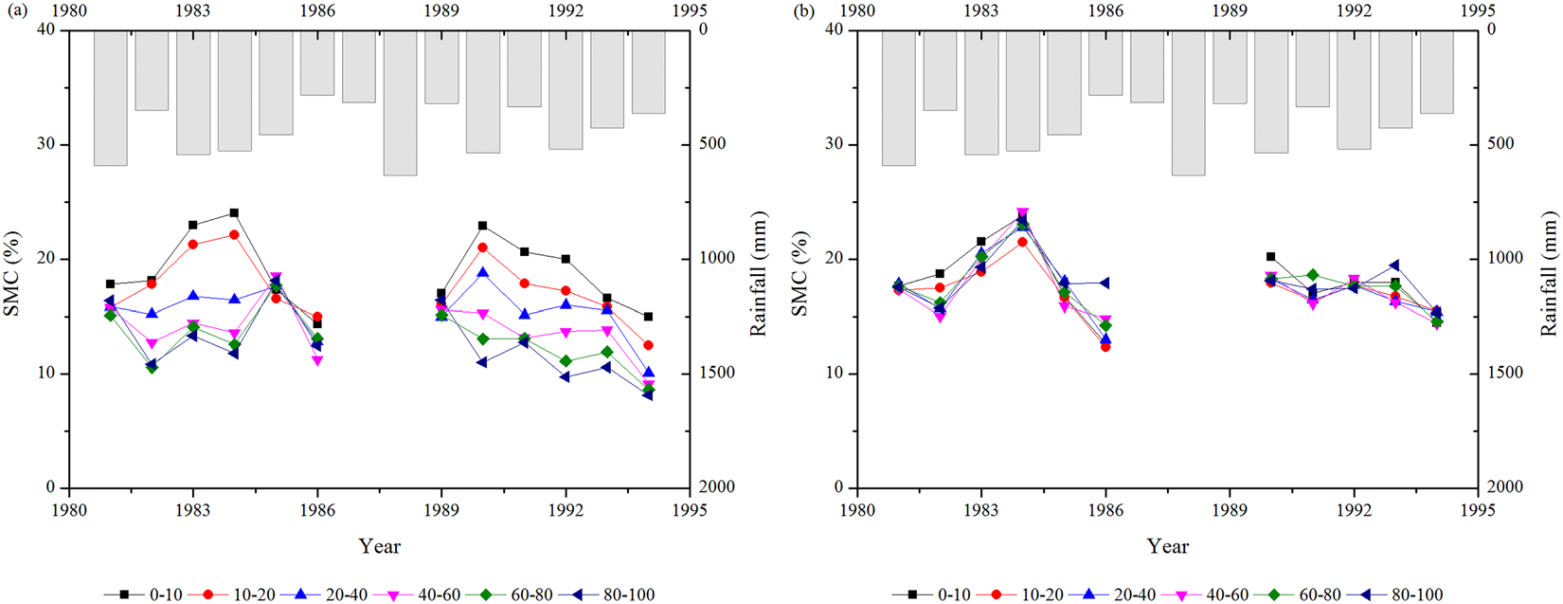
Annual variation in soil moisture content at different depths and total rainfall in the growth season in (**a**) Yangjiagou (forest land) and (**b**) Dongzhuanggou (grassland).

During 1981–1994, SMC in both the forest land and the grassland generally showed a slight decrease, which was consistent with the overall pattern of rainfall (Fig. 3).

### Seasonal changes in soil moisture content

**Figure 4:**
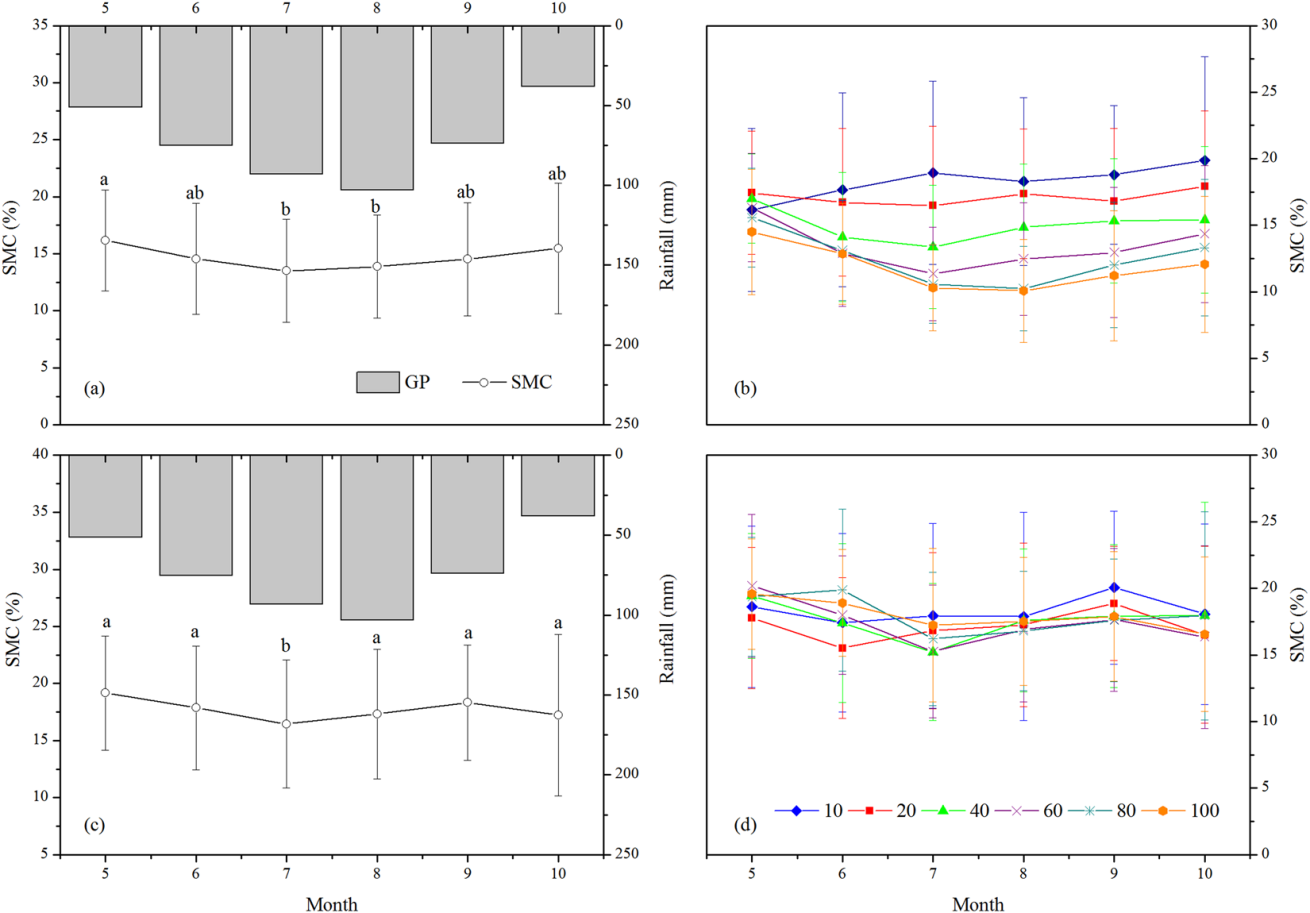
Seasonal changes in rainfall, soil moisture content in Yangjiagou (forest land) and Dongzhuanggou (grassland) watersheds. (**a**) Across the entire soil profile (0–100 cm depth) and (**b**) at different depths in the forest land; (**c**) across the entire soil profile (0–100 cm depth) and (**d**) at different depths in the grassland. Different letters indicate significant differences within the same depth at 0.05 level (i.e., P < 0.05). The values are mean ± standard error.

In Yangjiagou (the forest land), SMC of the entire soil profile decreased from May to July and then increased from July to October (Fig. 4a). However, the pattern of changes differed among the soil layers: in the top layer (0–10 cm), SMC generally increased during the growing season, whereas the deeper layers showed an initial decrease and then an increase from May to October (Fig. 4b). Moreover, the fluctuations in SMC were greater in the deeper layers than in the surface layer.

Compared to the forest land, the monthly variations in SMC in the grassland were less pronounced, but the overall pattern was the same, namely an initial decrease followed by increase during the growing season. Values of SMC for the entire soil profile were similar across the months, except for July (Fig. 4c) and those for each soil layer fluctuated only slightly (Fig. 4d).

### Changes in soil moisture content with annual rainfall

**Figure 5:**
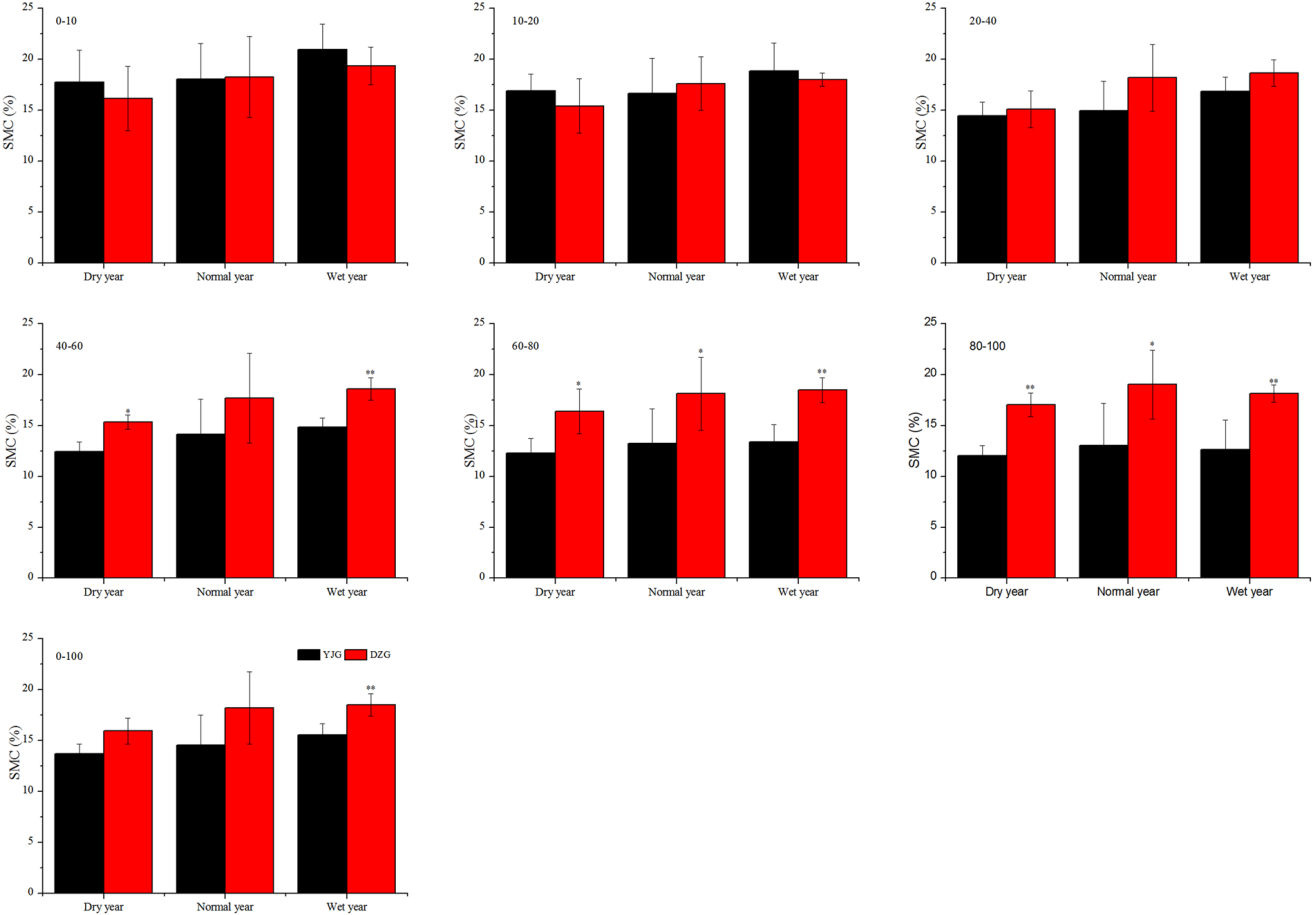
Mean (±standard deviation) soil moisture content at different depths in Yangjiagou (forest land) and Dongzhuanggou (grassland) watersheds in three types of rainfall years (normal, wet, and dry). An asterisk (*) indicates significant differences at 0.05 level.

In the upper layer (0–20 cm), SMC in the forest land was higher than that in the grassland in both dry years and wet years but slightly lower in normal years (Fig. 5). However, none of these differences were significant (P > 0.05). For the deeper layers (below 20 cm), SMC in the forest land was lower than that in the grassland in all the years; moreover, the differences were significant for layers deeper than 40 cm (P < 0.05) (Fig. 5). More important, the differences between the forest land and the grassland were more significant in wet years than in dry or normal years. Overall, SMC of the entire soil profile was lower in the forest land than that in the grassland; however, the difference was significant only in wet years (P < 0.05) (Fig. 5).

### Relationship between rainfall and soil moisture

Annual precipitation (AP) and precipitation during the growing season (GP) were significantly and positively correlated (*r* = 0.93; P < 0.01). In the forest land, both the correlations – between SMC and either AP or GP – weakened with depth. The correlation between SMC and AP was significant only for the 0–10 cm layer, whereas that between SMC and GP was significant for the 0–10 cm and the 20–40 cm layers (P < 0.05) (Table 3).

**Table 3:** Coefficients (Pearson’s *r*) of correlation between annual rainfall, rainfall in growing season, and soil moisture content of each layer in Yangjiagou (forest land) and Dongzhuanggou (grassland) watersheds.

Site		AP	GP	0–10	10–20	20–40	40–60	60–80
Yangjiagou (forest land)	GP	0.930**	1					
	0–10	0.590*	0.622*	1				
	10–20	0.449	0.532	0.950**	1			
	20–40	0.546	0.621*	0.683*	0.746**	1		
	40–60	0.398	0.463	0.285	0.343	0.823**	1	
	60–80	0.129	0.225	0.096	0.195	0.585*	0.873**	1
	80–100	0.022	0.103	−0.068	0.018	0.396	0.792**	0.950**
Dongzhuanggou (grassland)	0–10	0.591	0.676*	1				
	10–20	0.624*	0.709*	0.964**	1			
	20–40	0.648*	0.776**	0.907**	0.933**	1		
	40–60	0.555	0.691*	0.873**	0.851**	0.923**	1	
	60–80	0.498	0.626*	0.913**	0.893**	0.930**	0.930**	1
	80–100	0.242	0.441	0.681*	0.620*	0.733*	0.857**	0.834**

*Note*: AP: annual precipitation; GP: precipitation during growing season (May to October). N = 12 in the forest land and N = 11 in the grassland; **correlation significant at 0.01 level (two-tailed); *correlation significant at 0.05 level (two-tailed).

Compared to the relationships between SMC and either AP or GP in the forest land, those in the grassland were stronger. The correlation between SMC and AP was significant for the 0–40 cm layer, whereas that between SMC and GP was significant also for the 0–80 cm layer (P < 0.05). Moreover, the correlation between SMC and either AP or GP followed a unimodal curve, in that the highest correlation coefficient was for the 20–40 cm layer (Table 3). In addition, SMC of the different layers showed a strong correlation with their adjacent layers, and the correlation between the SMC of each layer in the grassland was markedly higher than that in the forest land (Table 3).

**Figure 6:**
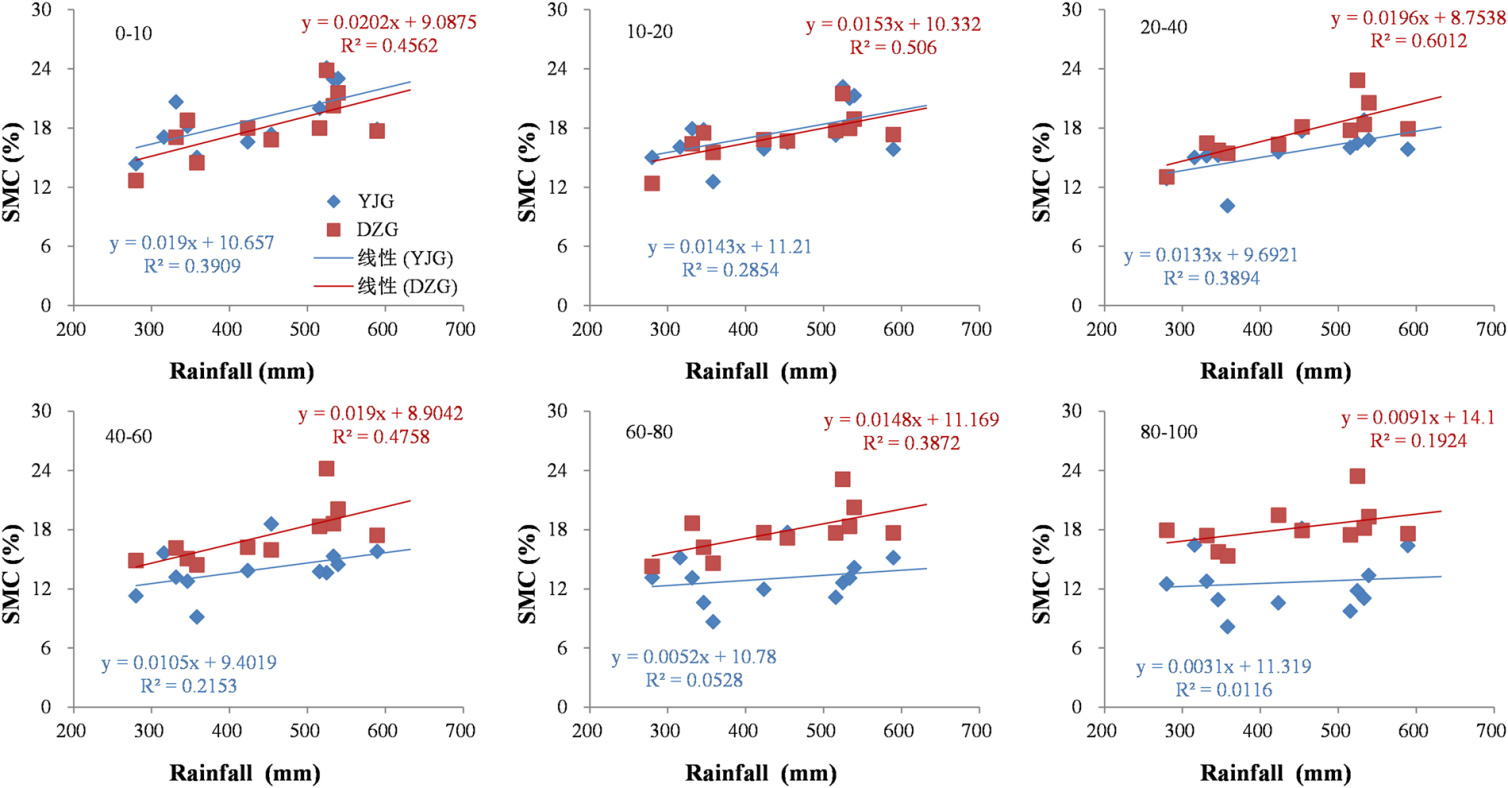
Relationships between rainfall during the growing season and soil moisture content at different depths in Yangjiagou (forest land) and Dongzhuanggou (grassland) water basin.

Regression analyses between SMC and GP indicated that precipitation during growing season could explain about 22–39% of the variation in SMC for the 0–60 cm layer, but no more than 5% in the 60–100 cm layer in the forest land (Fig. 6). In contrast, GP could explain about 46–60% of the variation in the 0–60 cm layer but only 39% in the 60–80 cm layer and only 19% in the 80–100 cm layer in the grassland (Fig. 6).

## Discussion

### Effect of vegetation types on soil moisture content

Soil is considered desiccated if SMC is between the permanent wilting point and 60% of field capacity, otherwise known as the stable field capacity (SFC)^13,16^. In the present study, the annual mean SMC of the entire soil profile (0–100 cm) was 14.7% in the forest land and 17.7% in the grassland (Table S1), whereas the SFC in the study area was lower, at 12.6%^28^. Therefore, neither afforestation nor natural revegetation had resulted in desiccated soil in the study area. However, SMC in the forest land was close to, or lower, than the SFC in some dry years (Table 4), which indicates that afforestation consumes more soil water than natural revegetation—a conclusion supported by earlier research^2,18,21,22^.

**Table 4:** Mean, standard deviation (SD), and coefficient of variation (CV) of soil moisture content up to a depth of 100 cm in Yangjiagou (forest land) and Dongzhuanggou (grassland) watersheds: 1981–1994.

Site		1981	1982	1983	1984	1985	1986	1989	1990	1991	1992	1993	1994	mean (%)
Yangjiagou (forest land)	mean (%)	16.0	13.5	16.2	15.5	17.8	12.9	15.8	16.0	14.7	13.9	13.6	10.0	14.7
	SD	0.9	3.3	4.1	5.2	0.7	1.3	0.8	4.7	3.2	3.9	2.4	2.7	2.8
	CV (%)	5.7	23.5	23.6	30.9	4.0	10.1	5.0	27.4	20.6	26.4	17.0	25.1	18.3
Dongzhuan-ggou (grassland)	mean (%)	17.6	16.2	20.1	23.2	17.2	14.5	/	18.5	17.1	17.8	17.4	14.9	17.7
	SD	0.2	1.4	0.9	0.9	0.8	2.1	/	0.8	0.9	0.3	1.2	0.5	0.9
	CV (%)	1.2	8.3	4.7	4.1	4.7	14.6	/	4.5	5.4	1.7	7.1	3.5	5.4

The difference of SMC were not significantly in upper layers (0–40 cm) whereas SMC was significant higher in deeper layers (40–100 cm) in grassland than that in forest land (P < 0.05) (Fig. 2). The differences of SMC along the soil profile may reflect the characteristic water consumption of these two vegetation types^2,29^. Plants lower SMC by taking up moisture from soil through their roots and releasing it into the atmosphere by transpiration through their leaves^19^. Species-specific transpiration and rooting depth are the main sources of variation in SMC^8^. The difference in the vertical distribution of SMC between the two watersheds indicates that the deeper roots of trees can obtain water from a depth of 100 cm and beyond in the forest land, whereas the shallow roots of grasses in the grassland are limited to a depth of 60 cm (Table S1). The pattern of distribution of SMC in the forest land and the grassland was basically consistent with the pattern of root distribution^30^. Grasses have shallower roots than do most trees, and more than 90% of the roots in temperate grassland are found within the top 60 cm of soil^31^. In contrast, *R. pseudoacacia* trees can extend their roots as deep as 190 cm^6^. Due to both grass and trees can take up water from the shallow root zone, the effects of vegetation types on soil moisture are greatly reduced in the upper layers^30^. However, for deeper layer, the limited depth to which rainfall can infiltrate the soil and the steady water consumption by roots, large quantities of water are thus lost from deep-rooted woody vegetation^32,33^. Yang *et al*.^3^ also found that no significant difference in near-surface soil moisture among the vegetation types but significant differences in the deep soil layers which supported our results.

Rainfall in the growing season accounted for 46–60% of the variation in soil moisture in the 0–60 cm layer in the grassland, but only 22–39% of that in the forest land (Fig. 6). The relationship between soil moisture and rainfall in the grassland was stronger than that in the forest land (Table 4; Fig. 6), which suggests that forests exercised their influence on SMC in more complex ways than grasslands did. Jin *et al*.^6^ reported that planting trees can have positive, negative, or negligible effects on SMC along rainfall gradient. On the one hand, forest land supports a composite, multi-layered vegetative structure comprising trees, shrubs, and herbs, whereas grasslands have only a single layer. Thus, the forest canopy intercepts most of the rainfall when the rainfall events are small, and the captured water evaporates directly; hardly any water infiltrates the soil in small rainfall events. Moreover, forest trees require more water to support their higher biomass, and the greater biomass results in greater evapotranspiration—the result is lower SMC^34^. On the other hand, forest trees form a thick layer of litter, which not only increases the water-holding capacity of soil, but also checks soil run-off more efficiently than grasses: such a reduction in run-off can be as high as 44% in the humid regions^35^; forests thus increase the amount of water that infiltrates the soil at big rainfall events.

### Seasonal change of SMC

Total SMC is determined by the net balance and interplay between rainfall, evapotranspiration, and run-off^4^. In the study area, because of the extensive vegetative cover, there has been almost no run-off over the last three decades. Thus, the increase in SMC in the 0–10 cm layer during the growing season in the forest land indicates that rainfall had played a dominant role in determining the level of soil moisture in the surface layer (Fig. 4b). Since the loss of soil moisture from the surface layer can be easily made up by more frequent but small rainfall events^9,30^, rainfall during the growing season could completely replenish the SMC. Moreover, the initial decrease in SMC followed by the increase from May to October in the deeper layers (below 10 cm) (Fig. 4b) indicates that evapotranspiration had regulated the effects of rainfall on soil moisture. Although the rainfall was higher in July and August than in any other month, the growth and metabolism of the trees were also the most vigorous in the two months: the time of optimal tree growth coincided with the onset of rains^36^. A large amount of soil moisture was probably lost through transpiration from trees with deeper roots^10^, thus leading to SMC of the study area being the lower in July and August (Fig. 4a). Compared to the forest land, the monthly variations in SMC in the grassland were smaller, but the overall pattern still showed a decrease followed by increase (Fig. 4c,d). Korres *et al*.^37^ also found that grassland had higher mean SMC and much lower variation in its values compared to forest land.

### SMC in different rainfall types

Rainfall has a major influence on SMC in many arid and semi-arid regions^38^, and changes in SMC after planting are largely governed by local rainfall^6,24^. In both the forest land and the grassland, SMC decreased slightly during the study period (1981–1994) and the decrease was consistent with the overall pattern of rainfall over the same period (Fig. 3). Plant species differ greatly in their response to the differences in rainfall and run-off, and these differences collectively can lead to temporal variation in SMC^3,39^. In the forest land, the decline in SMC was more marked than that in the grassland (Fig. 3). Researches have shown that trees consume more water than grasses do to sustain higher biomass and evapotranspiration, thereby depleting more water from soil^34,40^.

The widest differences in SMC between the forest land and the grassland were seen in the wet years, followed by the dry years, and the smallest differences were seen in the normal years. Evapotranspiration plays a key role in determining SMC of the Loess Plateau in China, since annual evaporation is about 2–10 times the rainfall^19^. In a wet year, although the higher rainfall increases SMC, evapotranspiration also increases significantly. Schipka *et al*.^41^ found that transpiration from the canopy of Central European beech forests increased linearly with rainfall when the annual rainfall was less than 700 mm. Wullschleger & Hanson^42^ reported that seasonal transpiration from the canopy of oak forests increased by 19% when rainfall increased by 33%, but decreased by as much as 30% when rainfall decreased by the same amount. In a forest land, a considerable proportion of rainfall is intercepted by trees and evaporates directly without ever reaching the ground, which means that much less water infiltrates the soil^34^. According to Jian *et al*.^5^, 21.1% of the total annual rainfall is intercepted by the canopy of *R. pseudoacacia* in the northern part of Loess Plateau in China^5^. In contrast, in grasslands infiltration is higher and faster, and evaporation is slower^20^. This observation echoes the findings of Garcia-Estringana *et al*.^30^, who reported that any increase in SMC during wet spells was more irregular and slower when the land was covered by trees, as in forests, than when it was covered by grasses.

In the dry or normal years, the mean rainfall in the study region was about 400–500 mm, which was enough to meet the normal growth requirements of native grasses^2,29^ but failed to sustain the normal growth of forest trees—in response, the trees ended up drawing water from the deeper layers^6,19^, a phenomenon that has been observed in other field studies as well. When water is in short supply in the shallow layer (<1 m), plant tap into the water resources of the deeper layer and then release the absorbed water into the shallow layer to sustain rapid growth^2,7^. Such replenishment of water in the shallow layer from the deeper layers in forest lands may have lowered the differences in SMC between the forest land and the grassland in the dry or normal years (Fig. 5).

### Implications for management

In our study, the twelve-year SMC observation data clearly indicated that neither the afforestation nor natural revegetation could induce the soil desiccation within the study area where the mean annual rainfall was 515 mm. Previous research also pointed out that afforestation was only recommended on the Loess Plateau where the mean annual rainfall was from 480 mm to 617 mm depending on the site conditions^2,6,43^. Therefore, it is suggested that afforestation would become a better option for the Loess Plateau only in areas with the annual rainfalls of more than 500 mm. Moreover, in any attempt for revegetation, the choice of tree species and planting densities should match the carrying capacity of the region’s water resources. For areas that have already formed a severe soil moisture deficit, converting forest to natural grassland could be an alternative approach to recover soil moisture and to avoid more serious ecological degradation.

Afforestation could be successful only in regions with adequate annual rainfalls. The mean annual rainfall have long been recognized as a prerequisite in afforestation^2,6^. However, there is great variation in inter-annual rainfall that the planted trees might die in the dry years which would lead to the failure of afforestation. Therefore, in order to avoid soil desiccation or the death of trees in the region, it is suggested that the annual rainfall in dry year rather than the mean annual rainfall should be considered as the lower limit in afforestation practice. In addition, rainfall during the growing season is a good explanatory variable in predicting the dynamics of surface soil moisture in the grassland but a poor predictor in explaining soil moisture changes in forestland of the Loess Plateau.

## Electronic supplementary material

Table S1

## References

[1] Legates DR (2011). Soil moisture: A central and unifying theme in physical geography. Prog. Phys. Geog..

[2] Deng L, Yan W, Zhang Y, Shangguan Z (2016). Severe depletion of soil moisture following land-use changes for ecological restoration: evidence from northern China. Forest. Ecol. Manag..

[3] Yang L, Wei W, Chen L, Chen W, Wang J (2014). Response of temporal variation of soil moisture to vegetation restoration in semi-arid Loess Plateau, China. Catena.

[4] Seneviratne SI (2010). Investigating soil moisture-climate interactions in a changing climate: A review. Earth-Sci. Rev..

[5] Jian S, Zhao C, Fang S, Yu K (2015). Effects of different vegetation restoration on soil water storage and water balance in the Chinese Loess Plateau. Agr. Forest Meteorol..

[6] Jin TT, Fu BJ, Liu GH, Wang Z (2011). Hydrologic feasibility of artificial forestation in the semi-arid Loess Plateau of China. Hydrol. Earth Syst. Sc..

[7] Prieto I, Ryel RJ (2014). Internal hydraulic redistribution prevents the loss of root conductivity during drought. Tree Physiol..

[8] Schume H, Jost G, Katzensteiner K (2003). Spatio-temporal analysis of the soil water content in a mixed Norway spruce (*Picea abies* (L.) Karst.) - European beech (*Fagus sylvatica* L.) stand. Geoderma.

[9] Vivoni ER (2008). Vegetation controls on soil moisture distribution in the Valles Caldera, New Mexico, during the North American monsoon. Ecohydrology.

[10] Wang S, Fu BJ, Gao GY, Yao XL, Zhou J (2012). Soil moisture and evapotranspiration of different land cover types in the Loess Plateau, China. Hydrol. Earth Syst. Sc..

[11] Jia, X., Shao, M. a., Zhu, Y. & Luo, Y. Soil moisture decline due to afforestation across the Loess Plateau, China. *J. Hydrol*. **546**, 113–122 (2017).

[12] Deng L, Shangguan Z, Sweeney S (2014). “Grain for Green” driven land use change and carbon sequestration on the Loess Plateau, China. Sci. Rep..

[13] Wang Y, Shao M, Zhu Y, Liu Z (2011). Impacts of land use and plant characteristics on dried soil layers in different climatic regions on the Loess Plateau of China. Agr. Forest Meteorol..

[14] Chen Y (2015). Balancing green and grain trade. Nat. Geosci..

[15] Shangguan Z, Zheng S (2006). Ecological properties of soil water and effects on forest vegetation in the Loess Plateau. Int. J. Sust. Dev. World.

[16] Yan W, Deng L, Zhong Y, Shangguan Z (2015). The characters of dry soil layer on the Loess Plateau in China and their influencing factors. Plos One.

[17] Chen L, Huang Z, Gong J, Fu B, Huang Y (2007). The effect of land cover/vegetation on soil water dynamic in the hilly area of the loess plateau, China. Catena.

[18] Wang L, Wang Q, Wei S, Shao M, Li Y (2008). Soil desiccation for loess soils on natural and regrown areas. Forest. Ecol. Manag..

[19] Wang Y, Shao M, Shao H (2010). A preliminary investigation of the dynamic characteristics of dried soil layers on the Loess Plateau of China. J. Hydrol..

[20] Wang S, Fu BJ, Gao GY, Liu Y, Zhou J (2013). Responses of soil moisture in different land cover types to rainfall events in a re-vegetation catchment area of the Loess Plateau, China. Catena.

[21] Zhang Y, Shangguan Z (2016). The change of soil water storage in three land use types after 10 years on the Loess Plateau. Catena.

[22] Yang L, Wei W, Chen LD, Mo BR (2012). Response of deep soil moisture to land use and afforestation in the semi-arid Loess Plateau, China. J. Hydrol..

[23] Zhang Y, Deng L, Yan W, Shangguan Z (2016). Interaction of soil water storage dynamics and long-term natural vegetation succession on the Loess Plateau, China. Catena.

[24] Liu BX, Shao MA (2016). Response of soil water dynamics to precipitation years under different vegetation types on the northern Loess Plateau, China. J. Arid Land.

[25] Jin Z (2016). Comparing watershed black locust afforestation and natural revegetation impacts on soil nitrogen on the Loess Plateau of China. Sci. Rep..

[26] Chen, L. Z. *Chinese Flora and Vegetation Geography*. (Science Press, 2014).

[27] Jin Z (2014). Natural vegetation restoration is more beneficial to soil surface organic and inorganic carbon sequestration than tree plantation on the Loess Plateau of China. Sci. Total Environ..

[28] Yang, W. Z & Shao, M. A. *Soil Water Research on the Loess Plateau*. (Science Press, 2000).

[29] Xiao L, Xue S, Liu GB, Zhang C (2014). Soil moisture variability under different land uses in the Zhifanggou catchment of the Loess Plateau, China. Arid Land Res. Manag..

[30] Garcia-Estringana P, Latron J, Llorens P, Gallart F (2013). Spatial and temporal dynamics of soil moisture in a Mediterranean mountain area (Vallcebre, NE Spain). Ecohydrology.

[31] Jobbágy EG, Jackson RB (2000). The vertical distribution of soil organic carbon and its relation to climate and vegetation. Ecol. Appl..

[32] Asner GP (2008). Invasive plants transform the three-dimensional structure of rain forests. P. Natl. Acad. Sci. USA.

[33] Zhang X (2017). Spatial variations and impact factors of soil water content in typical natural and artificial grasslands: a case study in the Loess Plateau of China. J. Soil Sediment..

[34] Jackson RB (2005). Trading water for carbon with biological sequestration. Science.

[35] Farley KA, Jobbágy EG, Jackson RB (2005). Effects of afforestation on water yield: a global synthesis with implications for policy. Glob. Change Biol..

[36] Piao SL (2003). Interannual variations of monthly and seasonal normalized difference vegetation index (NDVI) in China from 1982 to 1999. J. Geophys. Res..

[37] Korres W (2015). Spatio-temporal soil moisture patterns – A meta-analysis using plot to catchment scale data. J. Hydrol..

[38] Longobardi A (2008). Observing soil moisture temporal variability under fluctuating climatic conditions. Hydrol. Earth Syst. Sc. Discuss..

[39] Aranda I, Forner A, Cuesta B, Valladares F (2012). Species-specific water use by forest tree species: From the tree to the stand. Agr. Water Manag..

[40] Zhang L, Dawes WR, Walker GR (2001). Response of mean annual evapotranspiration to vegetation changes at catchment scale. Water Resour. Res..

[41] Schipka F, Heimann J, Leuschner C (2005). Regional variation in canopy transpiration of Central European beech forests. Oecologia.

[42] Wullschleger SD, Hanson PJ (2006). Sensitivity of canopy transpiration to altered precipitation in an upland oak forest: evidence from a long-term field manipulation study. Glob. Change Biol..

[43] Xu JX (2005). Threholds in vegetation-precipitation relationship and the implications in restoration of vegetation on the Loesee Plateau,China. Acta Ecol. Sin..

