# Supplementary material for: Comparing watershed afforestation and natural revegetation impacts on soil moisture in the semiarid Loess Plateau of China
Source: Sci Rep. 2018 Feb 14;8:2972. doi: 10.1038/s41598-018-21362-5 (PMC5813155; doi:10.1038/s41598-018-21362-5)
Supplement: Supplementary file 1 — Table S1 [file 41598_2018_21362_MOESM1_ESM.doc]

**Comparing watershed afforestation and natural revegetation impacts on soil moisture in the semiarid Loess Plateau of China**

Zongping Ren^1^, Zhanbin Li^1^, Xiaolu Liu^1^, Peng Li^1^, Shengdong Cheng^1^ & Guoce Xu^1^

^1^ State Key Laboratory Base of Eco-Hydraulic Engineering in Arid Area, Xi’an University of Technology, Xi’ an, 710048, China. Correspondence and requests for materials should be addressed to Z.P.R. (email:renzongping@163.com) or P. L. (email:lipeng74@163.com)

**Table S1**. Soil moisture content (%) at different depths in Yangjiagou (forest land) and Dongzhuanggou (grassland) watersheds.

| Site | Soil layer (cm) | Mean (%) | Median | Max. | Min. | SD | CV | n |
| --- | --- | --- | --- | --- | --- | --- | --- | --- |
| Yangjiagou (forest land) | 0–10 | 18.9 | 18.0 | 24.1 | 14.4 | 3.2 | 16.9 | 12 |
|  | 10–20 | 17.4 | 16.9 | 22.2 | 12.5 | 2.8 | 16.2 | 12 |
|  | 20–40 | 15.5 | 15.7 | 18.8 | 10.1 | 2.2 | 14.5 | 12 |
|  | 40–60 | 13.9 | 13.8 | 18.6 | 9.1 | 2.4 | 17.0 | 12 |
|  | 60–80 | 13.0 | 13.1 | 17.7 | 8.6 | 2.4 | 18.2 | 12 |
|  | 80–100 | 12.6 | 12.1 | 18.1 | 8.2 | 3.0 | 23.6 | 12 |
|  | 0–100 | 14.7 | 15.1 | 17.8 | 10.0 | 2.0 | 14.0 | 12 |
| Dongzhuanggou (grassland) | 0–10 | 18.1 | 18.0 | 23.8 | 12.7 | 3.1 | 17.1 | 11 |
|  | 10–20 | 17.1 | 17.3 | 21.5 | 12.4 | 2.2 | 13.0 | 11 |
|  | 20–40 | 17.5 | 17.8 | 22.8 | 13.0 | 2.6 | 15.0 | 11 |
|  | 40–60 | 17.4 | 16.2 | 24.2 | 14.4 | 2.9 | 16.4 | 11 |
|  | 60–80 | 17.8 | 17.7 | 23.1 | 14.3 | 2.5 | 13.9 | 11 |
|  | 80–100 | 18.2 | 17.9 | 23.4 | 15.3 | 2.1 | 11.8 | 11 |
|  | 0–100 | 17.7 | 17.4 | 23.2 | 14.5 | 2.4 | 13.6 | 11 |

*Note*: Soil moisture content was monitored during the growing season (May to October) each year from 1981 to 1994 (although the data were not available for 1987–1988 for the forest land and for 1987–1989 for the grassland.
